# Engineered small extracellular vesicles as a versatile platform to efficiently load ferulic acid *via* an “esterase-responsive active loading” strategy

**DOI:** 10.3389/fbioe.2022.1043130

**Published:** 2022-11-09

**Authors:** Fulong Man, Huaran Xing, Haoran Wang, Junfeng Wang, Rong Lu

**Affiliations:** ^1^ Marine College, Shandong University, Weihai, China; ^2^ Weihai Neoland Biosciences Co.,Ltd., Weihai, China

**Keywords:** esterase-responsive active loading, small extracellular vesicles, drug delivery, isolation and purification, ferulic acid esterase A, ferulic acid

## Abstract

As nano-drug carriers, small extracellular vesicles (sEVs) have shown unique advantages, but their drug loading and encapsulation efficiency are far from being satisfied, especially for the loading of hydrophilic small-molecule drugs. Inspired by the strategies of active loading of liposomal nanomedicines, pre-drug design and immobilization enzyme, here we developed a new platform, named “Esterase-responsive Active Loading” (EAL), for the efficient and stable drug encapsulation of sEVs. Widely used ferulic acid ester derivatives were chosen as prodrugs based on the EAL of engineered sEVs to establish a continuous transmembrane ion gradient for achieving efficient loading of active molecule ferulic acid into sEVs. The EAL showed that the drug loading and encapsulation efficiency were around 6-fold and 5-fold higher than passive loading, respectively. Moreover, characterization by nano-flow cytometry and Malvern particle size analyzer showed that differential ultracentrifugation combined with multiple types of membrane filtration methods can achieve large-scale and high-quality production of sEVs. Finally, extracellular and intracellular assessments further confirmed the superior performance of the EAL-prepared sEVs-loaded ferulic acid preparation in terms of slow release and low toxicity. Taken together, these findings will provide an instructive insight into the development of sEV-based delivery systems.

## 1 Introduction

Due to low bioavailability and toxic side effects, free drugs alone are often unable to meet the needs of clinical treatment. To solve this problem, Drug Delivery System (DDS) has emerged as a technology that can regulate the delivery rate of drugs, prolong the systemic circulation time, and deliver drugs to the desired target (Peer et al., 2007; Wang et al., 2021b). To date, many drug carriers, such as liposomes, nanoparticles and micelles, have been developed to ameliorate the shortcomings of therapeutic drugs (Ozpolat et al., 2014; Moosavian et al., 2021). However, these drug carriers accumulate outside the targets, which not only produces certain side effects on the human body but also depletes the amount of drug delivered to the desired location, resulting in a significant reduction in drug delivery efficiency to meet the expected clinical needs (Blanco et al., 2015; Wang et al., 2015). Therefore, there is an urgent need to find a more suitable drug carrier to eliminate the drawbacks associated with conventional synthetic nanocarriers.

Small extracellular vesicles (sEVs) are extracellular nanoscale vesicles with lipid-bilayer enclosures and biomolecule contents formed by most cells through a series of precise regulation processes such as “endocytosis-fusion-efflux”, which play an important role in intercellular information communication (Mathieu et al., 2019; Kalluri and LeBleu, 2020; van Niel et al., 2022). As novel nanocarriers, sEVs have shown great potential in drug delivery systems due to their excellent characteristics such as low immunogenicity, good biocompatibility, and intrinsic targeting (Abhange et al., 2021; Isaac et al., 2021). Up to now, there have been several sEV-based preclinical trials investigating the feasibility of delivering protein, RNA or other chemotherapeutic drugs to treat diseases (de Jong et al., 2019; Walker et al., 2019). Importantly, sEVs can cross biological barriers more easily, suggesting that sEVs should be more efficiently internalized than synthetic nanocarriers (Heusermann et al., 2016; Yang et al., 2020). Despite the desirable advantages of sEVs as drug carriers, the application of sEVs in drug delivery is just getting attention and some issues still need to be addressed: 1) which cells should be selected as the donors of sEVs; 2) how to modify the targeting of sEVs; 3) how to improve the drug loading efficiency of sEVs; and 4) how to achieve mass production of sEVs (Soekmadji et al., 2020; Wang et al., 2021a). Stable encapsulation of a large number of therapeutic molecules is a prerequisite for the use of sEVs as delivery vehicles (Jang et al., 2013; Lu and Huang, 2020). Current drug loading methods using sEVs are divided into two categories, pre-loading and post-loading (Shao et al., 2018; Herrmann et al., 2021; Kuriyama et al., 2021). Pre-loading is endogenous drug loading, relying on parental cells co-incubate with small molecule drugs or transfect with drug-encoding DNA, followed by loading of therapeutic drugs into sEVs through intracellular self-assembly behavior (Armstrong and Stevens, 2018; Liu and Su, 2019). This approach is used for drug loading by hijacking sEV biogenesis and is particularly suitable for loading biological therapeutics such as proteins or oligonucleotides. In contrast, post-loading is exogenous drug loading, that is, after sEVs are isolated from the parent cell cultures, the therapeutic drugs are loaded into sEVs using physical or chemical methods, such as ultrasound, electroporation and incubation (Kim et al., 2016; Goh et al., 2017). Nonetheless, whether loaded with biological drugs or chemical therapeutic agents, these methods shared a common problem: poor loading capacity, especially for the encapsulation of hydrophilic molecules (Donoso-Quezada et al., 2020; Chen et al., 2021). Therefore, the development of more advanced drug loading methods to improve the encapsulation of therapeutic agents is critical in sEV-based drug delivery systems.

In addition, a high yield of sEVs is essential for large-scale use or clinical studies. (Sharma et al., 2021; Pirisinu et al., 2022). Currently, a variety of sEVs isolation methods have been developed, such as differential ultracentrifugation, polymer precipitation, and size exclusion chromatography (Vader et al., 2016; Buschmann et al., 2021). However, these methods are either time-consuming and costly or furnishing sEVs low in concentration and purity, and it is very difficult to meet the requirements for industrial production of sEVs (Gardiner et al., 2016; Tieu et al., 2020). It has been shown that sEVs obtained using a combination of isolation methods are of better quality than one isolation method (Nordin et al., 2015; Shu et al., 2020). Therefore, the establishment of a more scientific and convenient sEV isolation process, which can rapidly purify and enrich high-quality sEVs from large volumes of cell cultures, will facilitate sEVs toward market translation and clinical applications.

Ferulic acid (FA) has various pharmacological effects such as anti-inflammatory, anti-oxidant, and anti-thrombotic, and has been widely used in the fields of medicine, food and cosmetics (Klepacka and Fornal, 2006; de Oliveira Silva and Batista, 2017; Antonopoulou et al., 2021). However, its hydrophilic characteristics limit its applicability in the pharmaceutical industry due to its low stability and low bioavailability (Han et al., 2019; Wang et al., 2021c). Until now, several types of nanocarriers loaded with FA have been reported for myeloid regeneration, wound healing, skin repair and neuroprotection (Cheng et al., 2011; Wu et al., 2014; Ou et al., 2021). Nevertheless, due to the feature of the carriers themselves, all of these carrier delivery strategies exhibited some degree of toxic side effects. Instead, due to the excellent characteristics of sEVs such as crossing biological barriers and biosafety, the drug delivery system composed of sEVs loaded with FA (sEVs-FA) can not only improve the stability of ferulic acid but also achieve well *in vivo* delivery. Therefore, it has become a promising direction to explore the application of sEVs-FA in skin repair.

**FIGURE 1:**
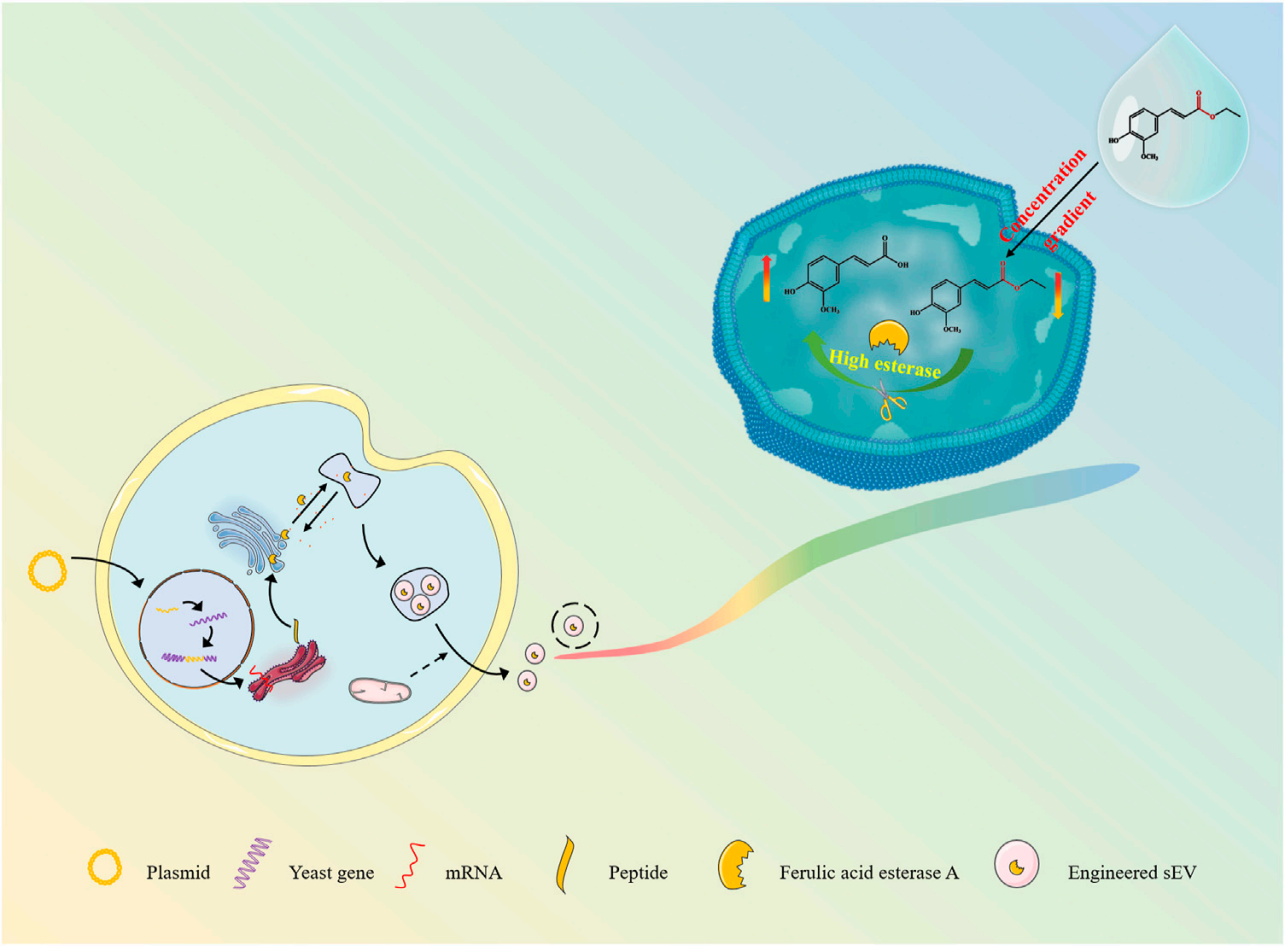
Scheme of sEV delivery system for efficient loading of hydrophilic drugs.

Inspired by the strategies of active loading of liposomal nanomedicines, pre-drug design and immobilization enzyme, here we are developing an esterase-responsive active loading (EAL) sEV-based drug loading platform for improved loading efficiency of the active molecules into sEVs, and establishing a scientific method for the large-scale isolation and purification of sEVs from cultures, as well as providing a delivery strategy for the application of ferulic acid in anti-aging and skin repair. (Figure 1). In the present study, we modified the *Pichia pastoris* X33 genome to incorporate the function of expressing ferulic acid esterase through modular genetic engineering techniques. The recombinant yeast expression product was subjected to structural analysis, and it was verified that the expressed ferulic acid esterase is biological activity. Engineered small extracellular vesicles (FaeA@sEVs) derived from recombinant yeast cells were isolated by differential ultracentrifugation combined with multiple types of membrane filtration methods. Corresponding FaeA@sEVs were characterized based on their morphology, particle size distribution and potential distribution. Furthermore, the storage stability at different temperatures, the hydrolysis function and the kinetic parameters of the enzymatic reaction of FaeA@sEVs were also investigated. Then, the loading efficiency of FaeA@sEVs for the water-soluble drug ferulic acid was determined by high-performance liquid chromatography (HPLC) and compared with blank small extracellular vesicles (X33@sEVs) to highlight the advantages of the EAL strategy. In addition, the physicochemical properties of engineered small extracellular vesicles loaded with ferulic acid (FaeA@sEVs-FA) were identified. Finally, the formulation process evaluation and cytotoxicity experiments involving FaeA@sEVs-FA were further conducted to evaluate the *in vitro* release characteristics and biosafety of FaeA@sEVs-FA, which laid the foundation for the subsequent development of FaeA@sEVs-FA into skin repair products.

## 2 Materials and methods

### 2.1 Cell and strain culture

Human embryonic kidney (HEK) 293T cells were purchased from Weihai Newland Biological Company and were cultured in RPMI-1640 containing 10% fetal bovine serum, penicillin (100 μg/ml), and streptomycin (100 μg/ml) at 37°C in a 5% CO_2_ humidified incubator; digested with 0.25% trypsin for passage. The *Escherichia coli* (*E. coli*) was cultured in an LB culture medium (37°C, 220 rpm). The *Pichia pastoris* X33 was cultured in a BMGY culture medium (30°C, 250 rpm).

### 2.2 Plasmid design

According to the ferulic acid esterase A (FaeA) gene sequence (XM_025603867.1) (Miaoling, Wuhan) and the multiple cloning site on plasmid pPICZA (Jinweizhi, Suzhou), two pairs of specific upstream and downstream primers (Jinweizhi, Suzhou) were designed to construct the coding DNA sequence of FaeA. Upstream primer 1 (FaeA-F-1): 5′-ATG​AAG​CAA​TTC​TCT​GCA​AAA​TAC-3′; Downstream primer 2 (FaeA-R-2): 5′-GCC​ACC​GCC​ACC​AGACCA​AGT​ACA​AGC​TCC​GCT-3′ (underlined as linker sequence); upstream primer 3 (FaeA-F-3): 5′-TCA​AAA​AAC​AAC​TAA​TTA​TTC​GAA​ACG​AGGAA​TTCATG​AAG​CAA​TTC​TCT​GCA​AAA​TAC​GC-3′ (underlined as EcoRI restriction site); downstream primer 4 (FaeA-F-4): 5′-GCT​AAA​ACT​CAA​TGA​TGA​TGA​TGA​TGA​TGGTC​GACGCC​ACC​GCC​ACC​AGA​C-3′ (underlined as Sal I restriction site).

The construction steps of the expression plasmid were as follows: In the first step, the FaeA gene with a linker sequence at the 3′ end was amplified by PCR from the pEnCMV-FAEA-3×FLAG plasmid (Miaoling, Wuhan) using the upstream primer 1 and the downstream primer 2; in the second step, the EcoR I restriction site, the Sal I restriction site and the 6xHis-tag were introduced through the upstream primer 3 and the downstream primer 4; in the third step, the expression vector pPICZA was digested with EcoR I and Sal I restriction enzymes, and the FaeA gene containing the 6xHis-tag was spliced into the expression vector by seamless cloning. As shown in Supplementary Figure S1.

### 2.3 Recombinant expression plasmid cloning, linearization and transformation of yeast

After two rounds of PCR reactions and a seamless cloning kit (Beyotime, Shanghai) ligation, the ligation mixture was obtained, as shown in Supplementary Table S2. The ligation mixture was subsequently electro-transferred into *E.coli* DH5α competent cells (Angyu, Shanghai) using electroporation. Transformed colonies were selected on LB agar medium containing Zeocin Selection Reagent (25 μg/ml) (Sangon, Shanghai). Bacteria liquid was performed with universal primers (5′AOX1: 5′-GAC​TGG​TTC​CAA​TTG​ACA​AGC-3′ and 3′AOX1: 5′-GCA​AAT​GGC​ATT​CTG​ACA​TCC-3′) of the pPICZA vector (Invitrogen, United States) were subjected to a polymerase chain reaction, and colonies containing recombinant vectors were screened. Positive colonies were collected and cultured overnight in LB medium for plasmid cloning. A high concentration of recombinant expression plasmid named pPICZA-FaeA-6H was obtained using plasmid mid-extraction and plasmid concentration methods.

The recombinant expression plasmid pPICZA-FaeA-6H was incubated at 37°C for 18 h with restriction endonucleases SacⅠ(Beyotime, Shanghai) for single-enzyme digestion linearization. Similarly, the linearized product was electro-transferred to *Pichia pastoris* X33 competent cells by electroporation. Transformed colonies were selected on a YPD agar medium containing Zeocin Selection Reagent (25 μg/ml). Colonies containing the recombinant vector were initially screened by fungal liquid polymerase chain reaction using universal primers of the pPICZA vector mentioned above. Further, the screened colonies are identified by genome sequencing, and the colonies with correct sequences are preserved as high-copy recombinant yeast. As shown in Supplementary Figure S3.

### 2.4 Expression and identification of exogenous protein

200 μL of high-copy recombinant yeast liquid was taken out of the refrigerator at -80°C, transferred into 10 ml BMGY medium, and cultured in a shaker at 250 rpm and 30°C for 24 h. The next day, the OD600 of yeast liquid was detected, and a proper amount of yeast liquid was added to about 200 ml BMGY culture medium. The initial OD600 of yeast liquid was controlled to be about 1, and the yeast liquid was cultured in a shaker at 250 rpm and 30°C for 24 h. On the third day, the yeast liquid obtained on the second day was centrifuged at 6,100 rpm for 5 min. The precipitate was transferred to a 200 ml BMMY culture medium, and it was cultured in a shaker at 250 rpm and 30°C for 24 h. On the fourth day, 5‰ methanol was added to the culture medium, that is, 1 ml methanol was directly added, and the culture was continued cultured in a shaker at 250 rpm and 30°C for 24 h. On the fifth day, the culture medium obtained on the fourth day was collected and centrifuged at 6,100 rpm for 5 min. The supernatant was used to extract the engineered small extracellular vesicles (FaeA@sEVs), and the precipitate was used to extract the exogenous protein expressed by recombinant yeast cells.

Take 2 ml of the above precipitate in a 4 ml EP tube, add 1 ml of *Pichia pastoris* X33 cell lysate (1 mM EDTA-2Na, 50 mM NaH_2_PO_4_, 5% glycerol) containing 1% protease inhibitor. After mixing, the mixture was placed in an ice bath for 30 min. The cell wall of the above suspension was broken by ultrasound with a cell breaker (Bannuo, Shanghai). The parameters of the cell breaker were as follows: power 120 W, 10 s interval for each cycle of ultrasound, and 60 cycles in total. After centrifugation at 6,100 rpm for 5 min, the supernatant named intracellular protein was transferred to a new EP tube. Intracellular protein concentration was detected by a protein analysis kit (Beyotime, Shanghai). As the exogenous protein feruloyl esterase is linked to the fusion protein 6xHis-tag, the expression of exogenous protein feruloyl esterase can be verified by western blot with 6xHis-tag antibody.

### 2.5 Determination of enzyme activity and its influencing factors

The enzymatic properties of intracellular proteins were investigated using a classical reaction system (Zhang et al., 2012): 800 μL of PBS (0.1×, pH 6.4) and 100 μL of ethyl ferulate (EF) solution were added to 2 ml EP tubes, mixed evenly, and incubated at 40°C for 10 min. After that, 100 μL enzyme solution at appropriate dilutions was added, sealed with a sealing membrane, and incubated in the dark at 40°C for 10 min. After the reaction was completed, 400 μL glacial acetic acid was added to quench the reaction immediately, and then passed through a 0.45-μm pore size PES filter membrane for HPLC analysis. The reaction solution with pre-added glacial acetic acid was used as a blank control. The specific chromatographic conditions were as follows: C18–4.6 mm × 250 mm, 1% glacial acetic acid: methanol = 70:30, detection wavelength for 313 nm, detection time for 25 min, flow rate for 1 ml/min, and column temperature for 30°C. One unit (U) of enzyme activity was defined as the amount of enzyme required to release 1 μmol ferulic acid per minute under the experimental conditions above.

The optimum reaction temperature of the enzyme was determined by measuring the enzyme activity at different temperatures (25–60°C) in phosphate buffer solution (PBS) with pH 6.4. To study the thermal stability of the enzyme, the residual activity was detected after incubation at different temperatures for 1 h. Similarly, PBS of different pH (pH 3.0–9.0) was prepared to determine the optimum pH of the enzyme. To test the pH stability of the enzyme, the residual activity of the enzyme was assayed after pre-incubation in the above buffer at 50°C for 1 h.

### 2.6 Isolation and purification of FaeA@sEVs

The collected recombinant yeast culture suspension (≥3 L) after induction and expression of recombinant yeast was poured into a sterile centrifuge bucket, and the supernatant was collected after centrifugation at 5,000 *g* for 45 min at 4°C. A vacuum filtration device was assembled and the supernatant was filtered with a 0.8-μm pore size PES membrane under the action of a vacuum pump to remove large cell debris and large impurities. Subsequently, the filtrate passed through a tangential flow filtration system (TFF) with a 100-kDa mPES hollow fiber membrane module to concentrate the sample solution to 300 ml and the permeate was discarded to remove small impurities such as interfering proteins. Then the concentrated sample solution was centrifuged at 15,000 g for 30 min at 4°C, and the obtained supernatant was successively filtered through a 0.8-μm and 0.45-μm pore size PES membrane. Next, the filtrate was purified by a sucrose cushion according to the volume ratio of 32.5 ml of filtrate per tube and 6 ml 0.971 M sucrose solution (Ramakrishnaiah et al., 2013) at 100,000 g for 70 min at 4°C. At the end of the centrifugation, 6 ml sucrose cushion at the bottom of each tube was removed and combined into a new centrifuge tube. Finally, the collected sucrose cushion was purified and concentrated in a 15 ml ultrafiltration tube (Millipore, United States) to remove sucrose at 4,000 g for 20 min at 4°C and the concentrated solution was the suspension of the FaeA@sEVs. The sEVs obtained from unmodified yeast in the same way were recorded as X33@sEVs and used as a blank control group.

### 2.7 Physicochemical characterization of FaeA@sEVs

To detect the particle concentration and particle size of FaeA@sEVs, the sEV solution was diluted with fresh PBS at appropriate multiples and assayed by nano-flow cytometry (NanoFCM, Xiamen). At the same time, the same sample solution was treated with 0.1% Triton X-100 (Sigma-Aldrich) to rupture the sEV membrane structure (Lorincz et al., 2020; Zhou et al., 2021). Changes in particle number of three sEVs before and after lysis with 0.1% Triton X-100 in Supplementary Table S4. The purity of sEVs was expressed by the ratio of the difference of the particle number concentration values before and after membrane rupture to that before membrane rupture. To compare the accuracy of different detection instruments and the stability of FaeA@sEVs, the particle size and zeta potential size of the FaeA@sEVs were further analyzed by a Malvern particle size analyzer (Malvern PANalytical, United kingdom). The BCA protein concentration determination kit was used to detect the protein concentration of the FaeA@sEVs. To detect the morphological characteristics of the FaeA@sEVs, 20 μL sEV suspension was dropped onto a copper mesh with a carbon-supported membrane. After adsorbing naturally for 5 min at room temperature, the excess liquid was removed using filter paper. Then 20 μL 2% phosphotungstic acid solution was added to the copper mesh and the membrane was stained for 3 min Afterward, the excess liquid was removed using filter paper and the copper mesh was placed under an incandescent lamp to dry naturally. Finally, the morphology of sEVs was observed under the transmission electron microscope (FEI, United States). In addition, western blot analysis confirmed the existence of ferulic acid esterase in FaeA@sEVs. X33@sEVs and FaeA@sEVs-FA were characterized in the same way as described above.

### 2.8 Enzyme activity of FaeA@sEVs

The enzyme activity of FaeA@sEVs and intracellular protein were measured according to the optimum temperature and pH of FaeA obtained above. The procedure was essentially the same as that described in section 2.5, except that the temperature was adjusted to 50°C and pH was adjusted to 4.0 in the enzymatic reaction conditions, and the reaction time was extended to 3 h for the FaeA@sEVs group.

### 2.9 Kinetic parameters of FaeA in free and FaeA@sEVs states

A series of reaction systems containing different concentrations of EF (substrate) were prepared, so that the final concentration of substrate in the system was set to 10.2–102 μg/ml. Then, 100 μL intracellular protein solution or FaeA@sEVs solution was added to the system, and the reaction was carried out at 50°C and pH 4.0 for 10 min or 3 h. After the reaction was completed, 400 μL glacial acetic acid was added to the system to quench the reaction. The concentrations of FA (product) in different substrate concentration systems were quantified by HPLC, and the corresponding enzyme activities were calculated. In the blank control groups, buffer solution, intracellular protein solution obtained from unmodified *Pichia pastoris* X33 and blank sEV solution were used instead of enzyme solution. Kinetic parameters (Km, Vmax, and Kcat) were estimated using the curve fitting to the Michaelis–Menten equation (Origin 2021).

### 2.10 Storage stability of FaeA@sEVs

X33@sEVs and FaeA@sEVs from the same batch were stored at three temperatures of 4°C, -20°C and -80°C for 1, 3, 6, 9, 12, 15 and 30 days, respectively. The particle size distribution and particle concentration of these samples was detected at the set time point by nano-flow cytometry.

### 2.11 Loading efficiency of engineered sEV-loaded active molecules

We previously investigated the effect of the mass ratio of FaeA@sEVs to EF on the drug loading efficiency. The results showed that the drug loading efficiency increases with the increase of EF input in a certain concentration range. Finally, we selected the condition that the mass ratio of FaeA@sEVs to EF is 1:2 to determine the advantage of drug loading efficiency of the EAL platform.

First, 100 μL EF (0.800 mg/ml) was added to 100 μL FaeA@sEVs (0.400 mg/ml). Then, 800 μL PBS (0.1×, pH 4.0) was added to the above-mixed system to constitute the reaction system. The reaction system was sealed with a sealing film and incubated in a water bath at 50°C for 3 h. After co-incubation, the enzyme reaction was immediately terminated on ice, and the whole reaction system was subsequently incubated at 4 °C for 0.5 h to restore the stability of the sEV membrane. Then, the reaction mixture was transferred to a 100-kDa ultrafiltration tube followed by the addition of 1 ml PBS and then centrifuged at 4,000 g for 10 min at 4°C to remove unloaded free drug molecules. Subsequently, the liquid in the inner tube (T_in_) of the ultrafiltration tube (FaeA@sEVs-FA) and the liquid in the outer tube (T_out_) of the ultrafiltration tube (free drug) were collected separately in new 1.5 ml EP tubes. The volume of T_in_ and T_out_ was recorded. An equal volume of methanol was added to the T_in_ and sonicated for 1 h in a water bath to break the membrane structure of the FaeA@sEVs-FA to release the internal drug. At the same time, the free drug in the T_out_ was diluted by a certain multiple. Finally, the samples of T_in_ and T_out_ were analyzed by HPLC for the content of EF and FA. The specific chromatographic conditions were as follows: C18–4.6 mm × 250 mm, 1% glacial acetic acid - methanol (0 min: 40–60, 15 min: 50–50, 18 min: 40–60) as mobile phase, detection wavelength for 325 nm, detection time for 18 min, and column temperature for 30°C. The drug loading efficiency of the FaeA@sEVs was calculated by converting EF into FA content using n (EF): n (FA) = 1:1. The loading efficiency was expressed in terms of drug loading and encapsulation efficiency:

$$\%Drug\;loading=\frac{amount\;of\;drug\;in\;sEVs}{total\;amount\;of\;protein\;in\;sEVs}\times 100\%$$

$$\%Encapsulation\;efficiency=\frac{amount\;of\;drug\;in\;sEVs}{amount\;of\;drug\;added}\times 100\%$$

The same process was used to analyze the drug loading efficiency of X33@sEVs loaded EF, and the results were used as a blank control group. In addition, since EF is hydrolyzed to an equal amount of FA by the action of esterase, the drug loading efficiency of EF will eventually be calculated in the form of FA. Using the same method as described above, EF was replaced with FA to explore whether loading FA with FaeA@sEVs and X33@sEVs had an impact on the results of assessing loading EF.

### 2.12 *In vitro* drug release pattern of FaeA@sEVs-FA

Precisely aspirate 100 μL FaeA@sEVs-FA and 900 μL PBS into a dialysis bag (MW = 8,000). After tying the ends of the bag tightly, the dialysis bag was placed in 8 ml PBS (pH 7.4). The temperature was set at 32 ± 0.5°C, and the stirring speed was set at 200 r/min. 1 ml of release medium was taken from the outside of the dialysis bag at 10, 30, 60, 90, 120, and 150 min, while an equal volume of blank release medium was added at the same temperature. The contents of EF and FA in the release medium at different moments were measured according to the method described in section 2.5. The cumulative release Q was calculated by converting EF into FA content using n (EF): n (FA) = 1: 1.

### 2.13 Cytotoxicity analysis of FaeA@sEVs-FA

The cytotoxicity of free FA, FaeA@sEVs-FA, FaeA@sEVs and X33@sEVs at different concentrations was assessed by CCK-8 assay. HEK 293T cells were seeded at 1×10^4^/well in 96-well plates and cultured for 24 h. Then free FA solution and sEV with the concentration of 0.1–10.0 μM were added to the cells, and incubation was carried out for 24 h. All samples were diluted in complete medium. The concentration of FA in FaeA@sEVs-FA was the same as that of the free FA, and the particle concentration of FaeA@sEVs and X33@sEVs was the same as that of the FaeA@sEVs-FA. The cells were incubated with 10 μL CCK-8 reagent in the dark for 2 h. The cells were immediately placed in a microplate reader (Bio-Tek, United States) and the absorbance value at 450 nm was measured. The measurement was repeated three times. Cell viability (%)=(A2-A0)/(A1-A0)×100%, where A0 referred to the absorbance of the well containing medium and CCK-8 solution without cells, A1 referred to the absorbance of the well containing cells and CCK-8 solution without drug, and A2 referred to the absorbance of well containing cells, drug and CCK-8 solution.

### 2.14 Statistical analysis

Above experimental data were repeated three times (*n* = 3) and analyzed by GraphPadPrism 8.0 and Origin 2021 for data analysis, statistical analysis and image processing. The measurement data were expressed as mean ± standard deviation (Mean ± SD), and the data between groups were analyzed by one-way ANOVA to assess the difference between various groups: *∗p* < 0.05, *∗∗p* < 0.01, *∗∗∗p* < 0.001.

## 3 Results and discussion

### 3.1 Clonal expression and enzymatic properties of FaeA gene

**FIGURE 2:**
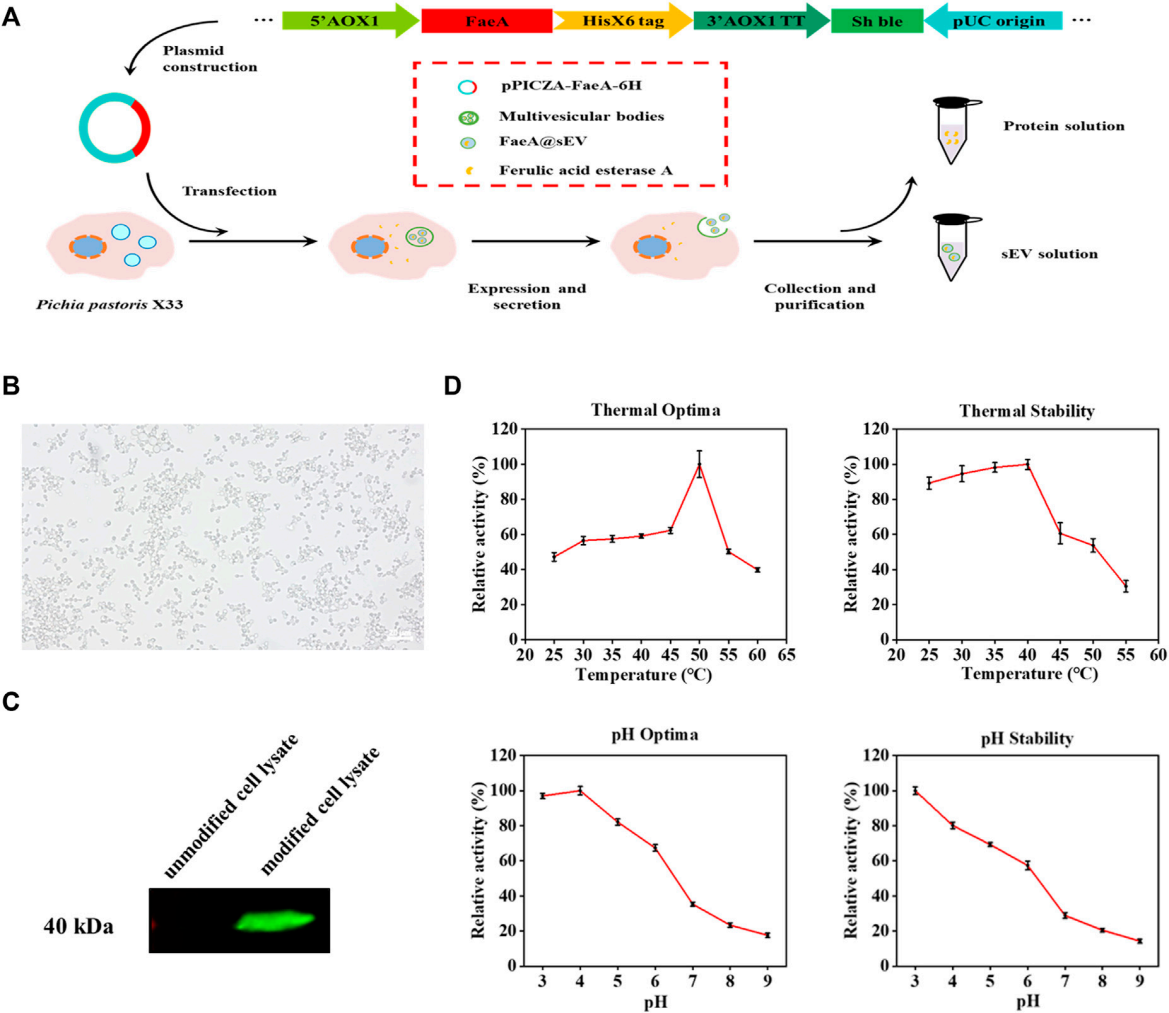
Clonal expression and enzymatic properties of FaeA gene. **(A)** The construction process of recombinant yeast expressing FaeA gene. **(B)** Inverted microscope imaging of how recombinant yeast grows. **(C)** Identification of FaeA in modified cell lysates by western blotting. **(D)** The enzymatic properties of FaeA. (*n* = 3, mean ± SD).

In this experiment, we modified the genome of *Pichia pastoris* X33 by modular genetic engineering techniques, and successfully obtained an engineered strain capable of expressing the exogenous protein FaeA (Figure 2A). The recombinant yeast growth state was observed under an inverted microscope (Caisi, China), and the result showed that the recombinant yeast divided in a typical budding manner (Figure 2B). The results of western blot analysis of the intracellular protein from recombinant yeast confirmed that recombinant yeast was able to express the exogenous protein FaeA with an apparent molecular weight of 40.0 kDa (Figure 2C). The apparent molecular weight was larger than the theoretical value of 32.0 kDa, which may be due to post-translational modifications such as phosphorylation and glycosylation of FaeA during recombinant yeast expression. Software NetPhosYeast 1.0, NetNGlyc 1.0 and NetOGlyc 4.0 (http://www.cbs.dtu.dk/services/) were used to predict the phosphorylation and N and O-glycosylation sites of the FaeA gene, respectively. The results showed the presence of an O-glycosylation site at amino acid position 272. Further analysis of the enzymatic properties of FaeA showed that the optimum temperature was 50°C, and the enzyme remained stable below 40°C; the optimum pH was 4.0, and the enzyme was more stable between pH 3.0 and 5.0 (Figure 2D).

### 3.2 Isolation and characterization of FaeA@sEVs

**FIGURE 3:**
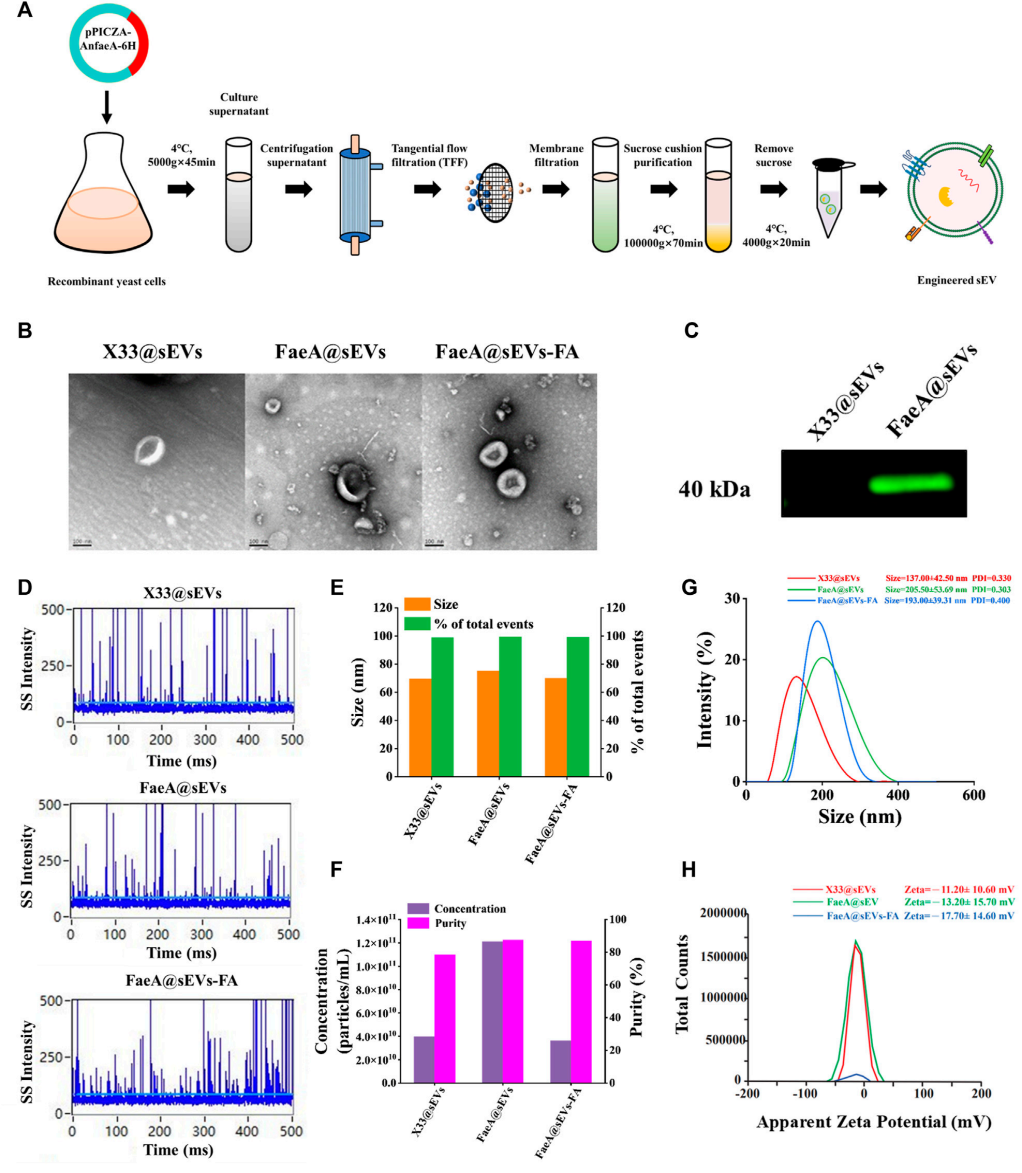
Isolation and characterization of sEVs. **(A)** Schematic protocol of isolation and purification of sEVs. **(B)** TEM images of three sEVs, scale bars represent 100 nm. **(C)** Identification of FaeA in engineered sEVs by western blotting. **(D)** Representative SS signal of three sEVs by nano-flow cytometry. **(E)** Particle size distribution and event percentage of three sEVs by nano-flow cytometry. **(F)** Particle concentration and purity of the three sEVs were measured by nano-flow cytometry. **(G)** Particle size distribution and PDI values of three sEVs by Malvern particle size analyzer. **(H)** Zeta potential distribution of three sEVs by Malvern particle size analyzer. (*n* = 3, mean ± SD).

Application of sEVs as nano-drug carriers requires high purity, otherwise their drug loading capacity or physiological processes *in vivo* will be compromised (Batrakova and Kim, 2015; Hettich et al., 2020). Additionally, rapid and large-scale production of sEVs is critical to extending their practical application including the need for clinical applications (Zhang et al., 2021). Hence, we performed differential ultracentrifugation combined with multiple types of membrane filtration methods to isolate and purify FaeA@sEVs from large volumes of recombinant yeast culture supernatant (Figure 3A), and characterized their morphology, particle size distribution, zeta potential distribution and concentration. Notably, due to the density of sEVs of 1.13–1.19 g/cm^3^ (van der Pol et al., 2012; Xie et al., 2019), we used a 0.971 M sucrose cushion in the isolation process to obtain high-quality FaeA@sEVs. Under transmission electron microscopy, we observed that the FaeA@sEVs exhibited a typical saucer-like and relatively clean background (Figure 3B). This low background without contamination by other impurities indicated that FaeA@sEVs were of high purity. To verify the presence of FaeA in the FaeA@sEVs, we performed a western blot analysis, and the result confirmed that FaeA was successfully loaded into the sEVs (Figure 3C). The complete western blot image in Supplementary Figure S5.

Since transmission electron microscopy has disadvantages such as time-consuming, limited field of view (Lai et al., 2022), and possible imaging artifacts, we further employed nano-flow cytometry and Malvern particle size analyzer to fully reveal the heterogeneity of FaeA@sEVs. Figure 3D showed that the side scatter (SS) signal occurred when a single particle passed through the tightly focused laser beam of nano-flow cytometry in sequence. Figure 3E displayed the particle size of X33@sEVs and FaeA@sEVs were 69.48 ± 11.91 nm and 71.91 ± 14.59 nm, respectively. The particle size distribution diagrams of three sEVs were included in Supporting Information, see Supplementary Figure S6. Figure 3F reflected the particle concentration of X33@sEVs and FaeA@sEVs were 3.98E+10 particles/mL and 1.21E+11 particles/mL, respectively. The change in particle number after triton treatment represented the purity of the sEVs (Lim et al., 2022), and the result showed that the purity of the FaeA@sEVs was close to 90%. Nano-flow cytometry could better reflect the physicochemical properties of a single particle, and these results also suggested that the engineered operation did not lead to significant differences in the morphology and particle size distribution of the sEVs.

Meanwhile, the results of the Malvern particle size analyzer showed that sample particles of single-peaked distribution. The particle size for X33@sEVs and FaeA@sEVs was 137.00 ± 42.50 nm and 205.50 ± 53.69 nm, respectively (Figure 3G). The particle size increased after the modification, which may be influenced by the content FaeA. Figure 3H suggested that the average potential and PDI values of X33@sEVs were -11.20 ± 10.60 mV and 0.330, respectively, while the average potential and PDI values of FaeA@sEVs were -13.20 ± 15.70 mV and 0.303, respectively. This indicates that the FaeA@sEVs have a more stable dispersion state. Due to the limitations of the experimental conditions, the Malvern particle size system is more widely applicable compared to the nano-flow cytometry system. Above sEV characterization experiments indicated inconsistent results of the two detection systems, which may be related to the detected sample volumes. The relatively larger volume of samples detected by the Malvern particle size analyzer presented interference between particles, which leads to a larger detection of sEV hydrodynamic diameter. In conclusion, the nano-flow cytometry provides more accurate results for the identification of sEVs. However, the Malvern particle size analyzer can easily measure the dispersion state of sEVs.

Further, the results of the characterization of the FaeA@sEVs-FA were as follows. Transmission electron microscopy showed typical saucer-like morphology of the FaeA@sEVs-FA (Figure 3B). The results of nano-flow cytometry showed that after loading drug FaeA@sEVs-FA particles displayed a single-peaked distribution with a mean particle size of 70.01 ± 10.92 nm, which was not significantly different from that before the loading (Figure 3E). The results of the Malvern particle size analyzer showed that the average particle size of the FaeA@sEVs-FA was 193.00 ± 39.31 nm, and the average potential of -17.70 ± 14.60 mV and PDI values of 0.400, as shown in Figures 3G,H. These results indicated that there was no significant change in the morphology and particle size of sEVs before and after loading the drug, and the FaeA@sEVs-FA was more dispersed and stable. We speculated that the addition of the FA may have stabilized the membrane structure of the sEVs. The characterization data of FaeA@sEVs-FA are summarized in Supplementary Table S7.

### 3.3 Esterase hydrolysis and storage stability of FaeA@sEVs

**FIGURE 4:**
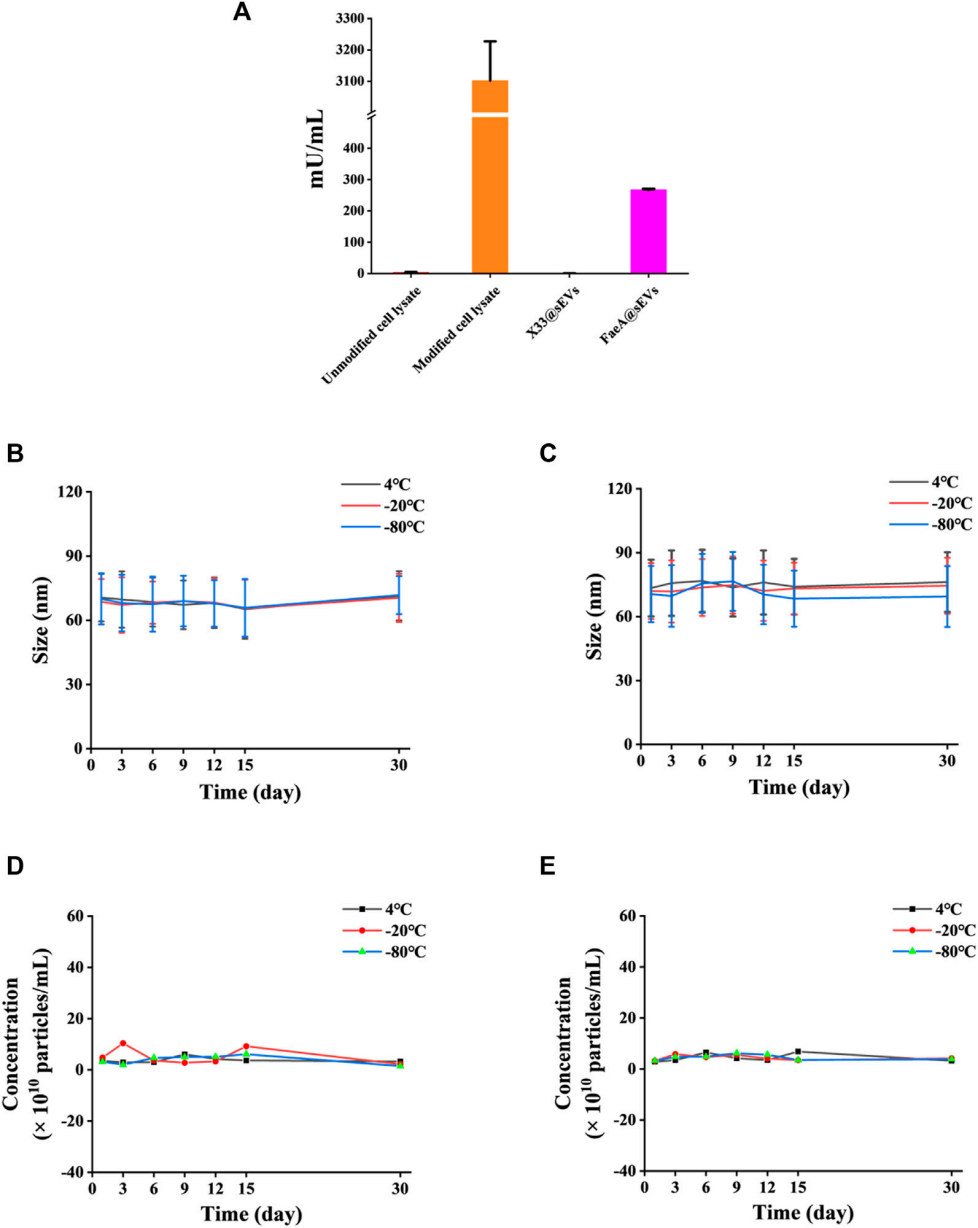
Esterase hydrolysis and storage stability of FaeA@sEVs. **(A)** Enzymatic activity of intracellular proteins and FaeA@sEVs. **(B)** Changes in the particle size distribution of X33@sEVs stored at 4, −20 and −80°C within 30 d. **(C)** Changes in the particle size distribution of FaeA@sEVs stored at 4, −20 and −80°C within 30 days **(D)** Changes in the particle concentration of X33@sEVs stored at 4, −20 and −80°C within 30 d. **(E)** Changes in the particle concentration of FaeA@sEVs stored at 4, −20 and −80°C within 30 d (*n* = 3, mean ± SD).

To further verify whether the FaeA@sEVs possess esterase hydrolysis, the enzymatic activity of FaeA@sEVs using EF as substrate was measured, and it was found as 267.63 mU/mL. In addition, the enzymatic activity data for intracellular proteins and sEVs indicated that FaeA maintains high hydrolytic capacity after being successfully incorporated into sEVs, as shown in Figure 4A. It has been reported that the physicochemical properties of mammalian-derived sEVs have not changed much when stored at 4°C for 2 weeks and can even be stored longer unchanged at -80°C (Yuan et al., 2021; Gelibter et al., 2022). However, the storage stability of yeast-derived sEVs has not been reported in the literature. We used a nano-flow cytometry to determine the change in particle size of X33@sEVs and FaeA@sEVs stored at different temperatures for several days. Figures 4B–E showed that the particle size and particle concentration of X33@sEVs and FaeA@sEVs did not change significantly within 30 days at 4°C, -20°C and -80°C, which indicated a wide range of storage conditions for the FaeA@sEVs and suggested that FaeA did not affect the storage conditions of the sEVs. Since the enzyme is usually kept at low temperatures, we have chosen to store the sEV suspensions at -80°C.

### 3.4 Kinetic parameters of FaeA in different states

The affinity and reaction rate of FaeA for the substrate were different in the naked and encapsulated states. sEVs as an encapsulated carriers affect the reaction rate of FaeA. To better understand the enzymatic activity of the FaeA@sEVs, we determined the kinetic parameters (Km, Vmax and Kcat) for the reaction of FaeA on EF in both states, and the results were shown in Table 1. The results displayed: intracellular protein (Vmax of 1,340.94 ± 489.12 U/mg) > FaeA@sEVs (Vmax value of 12.80 ± 3.88 U/mg) and intracellular protein (Km of 0.17 ± 0.09 mM) > FaeA@sEVs (Km of 0.10 ± 0.05 mM). Smaller Km values indicated a higher affinity of the enzyme for the substrate (Dulong et al., 2018; Schmitz et al., 2022). These data suggested that the immobilized enzyme system, which is formed by the method of FaeA being loaded into engineered sEVs, has an increased affinity for EF. However, in terms of the Kcat/Km ratio, the enzymatic reaction efficiency of the FaeA@sEVs was approximately 50-fold lower than that of the intracellular protein. This pattern is consistent with the characteristics of embedded immobilized enzymes (Grant et al., 2018), and suggests that FaeA is encapsulated inside the sEVs rather than adhering to the surface of the sEVs.

**TABLE 1:** Kinetic parameters of FaeA in different states.

	Km	Vmax	Kcat	Kcat/Km	Enzyme activity
	mM	U·mg^−1^	s^−1^	mM^−1^·s^−1^	mU·mg^−1^
Intracellular protein	0.17 ± 0.09	1,340.94 ± 489.12	1,570.19	9,236.40	148.47 ± 1.23
FaeA@sEVs	0.10 ± 0.05	12.80 ± 3.88	19.02	190.19	4.09 ± 0.07

### 3.5 Loading efficiency of FaeA@sEVs for active molecules

**FIGURE 5:**
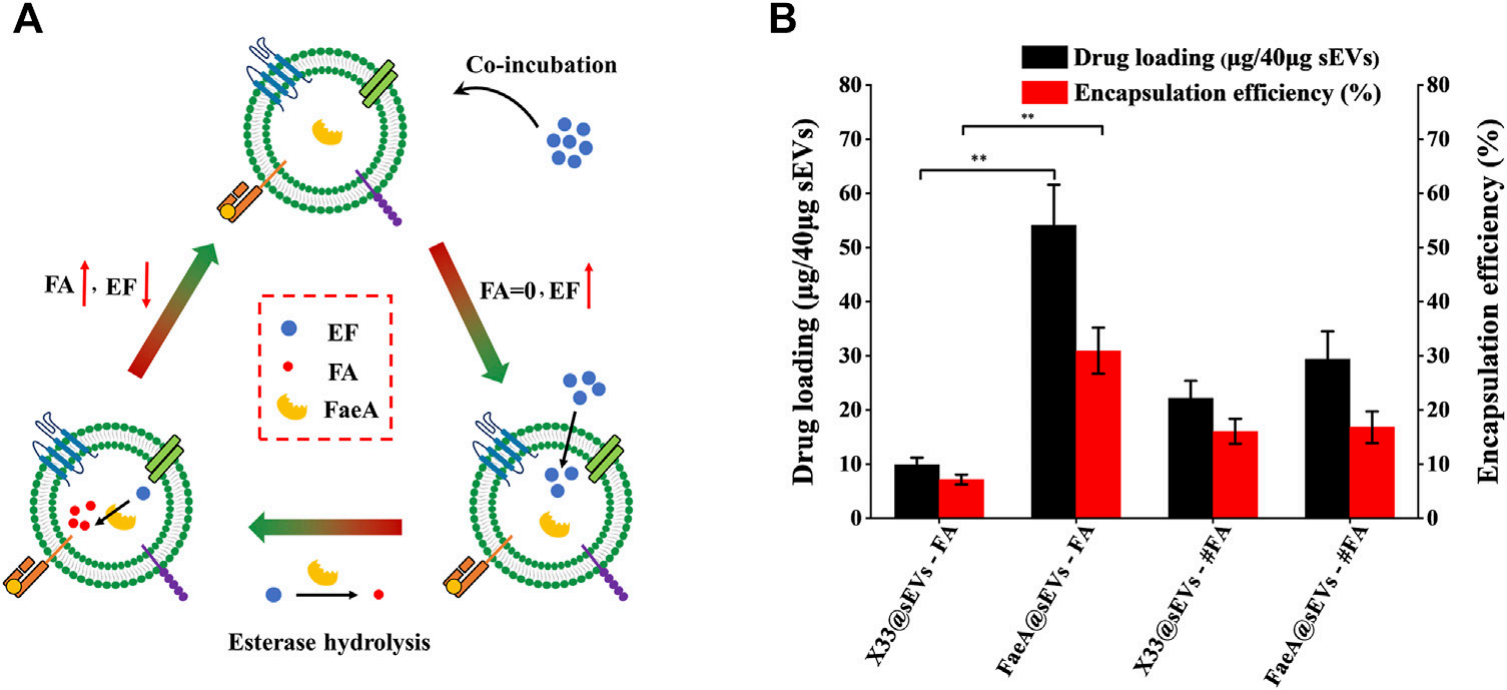
Loading efficiency of FaeA@sEVs loaded with active molecules. **(A)** Schematic representation of the EAL platform. When the EF with high concentration from the outside of the membrane to the inside of the membrane, it will undergo esterase hydrolysis to produce FA under the action of FaeA. This step is equivalent to closing the “switch” for the balance of EF concentration between the two sides of the membrane. As long as EF is present outside the membrane, it will enter the membrane depending on the concentration difference between the two sides of the membrane and release FA. At the same time, the ion trapping effect of biofilm can block the outward leakage of FA, thus indirectly achieving the efficient loading of FA in sEVs. **(B)** Loading efficiency of engineered sEVs loaded with active molecules. Note: X33@sEVs-#FA and FaeA@sEVs-#FA represent the drug loading efficiency obtained by replacing the loaded drug EF with FA, respectively. (*n* = 3, mean ± SD, **p < 0.01)

The principle of the co-incubation method is that the drug enters into the lipid bilayer of sEVs through hydrophobic intercalation, and its transport power is determined by the difference in the concentration gradient of the drug between the two sides of the membrane. The higher the concentration difference between the two sides of the membrane, the higher the transport power (Haney et al., 2015; Zhong et al., 2021). The process of the EAL is as follows: when the EF with high concentration from the outside of the membrane to the inside of the membrane, it will undergo esterase hydrolysis to produce FA catalyzed by FaeA. This step is equivalent to closing the “switch” for the balance of EF concentration between the two sides of the membrane. As long as EF is present outside the membrane, it will enter the membrane based on the concentration difference between the two sides of the membrane and release FA. At the same time, the ion trapping effect of biofilm can block the outward leakage of FA (Sun et al., 2021), thus indirectly achieving the efficient loading of FA in sEVs (Figure 5A).

Based on the previous methods for detecting FA content (Khezeli et al., 2016; Oliveira et al., 2019), we successfully obtained a chromatogram of EF and FA standards by HPLC (Supplementary Figure S8). By analyzing the change in the amount of the enzymatic action substrate (EF) and the hydrolysis product (FA), the amount of the final active component in the FaeA@sEVs can be determined according to the formula that 1 mol of EF produces 1 mol of FA. First, to find the optimal mass ratio of sEVs to the drug, we tested five combinations of mass ratios. The results showed that the highest drug loading of sEVs was achieved at a mass ratio of 1:2 between sEVs and EF (Supplementary Figure S9). Under the same conditions described above, we obtained the drug loading efficiency of X33@sEVs for EF, and the drug loading efficiency of X33@sEVs and FaeA@sEVs for FA (the molar amount of FA was equal to that of EF). The results showed that the EAL platform could significantly increase the drug (ferulic acid) loading (from less than 10% to nearly 60%) and encapsulation efficiency (from less than 8% to nearly 40%) of the drug active molecule ferulic acid (Figure 5B; Table 2 and Supplementary Table S10). Remarkably, since loading ferulic acid does not initiate the esterase response, the drug loading efficiency of the EAL platform is approximately 2-fold higher than using X33@sEVs (X33@sEVs-#FA) or FaeA@sEVs (FaeA@sEVs-#FA) for loading an equivalent amount of ferulic acid. The data above suggest that the EAL platform can significantly improve the drug loading and encapsulation efficiency of sEVs for water-soluble active drugs. Although classical drug loading methods, such as ultrasonication, electroporation and freeze-thaw cycles, could also improve drug loading to some extent, they may cause damage to the membrane structure of sEVs (Nasiri Kenari et al., 2020). In addition, some studies have shown that electroporation could lead to aggregation and precipitation of therapeutic drugs, which can lead to misinterpretation of drug loading efficiency (Kooijmans et al., 2013). The EAL platform provides drug loading power *via* biocatalysis and could protect the active drugs and carriers from damage by external mechanical conditions. Therefore, the EAL platform developed in this study has mild conditions and could maintain the morphology of sEVs well compared to other widely used drug loading methods, thus achieving a good loading efficiency.

**TABLE 2:** Loading efficiency of sEVs for active molecules.

Group	Drug loading (μg/40 μg sEVs)	Encapsulation efficiency (%)
X33@sEVs-FA	9.92 ± 1.28	7.18 ± 0.92
FaeA@sEVs-FA	54.10 ± 7.47**	30.96 ± 4.28**
X33@sEVs-#FA	22.19 ± 3.18	16.07 ± 2.31
FaeA@sEVs-#FA	29.39 ± 5.15	16.82 ± 2.95

### 3.6 *In vitro* drug release pattern and cytotoxicity of FaeA@sEVs-FA

**FIGURE 6:**
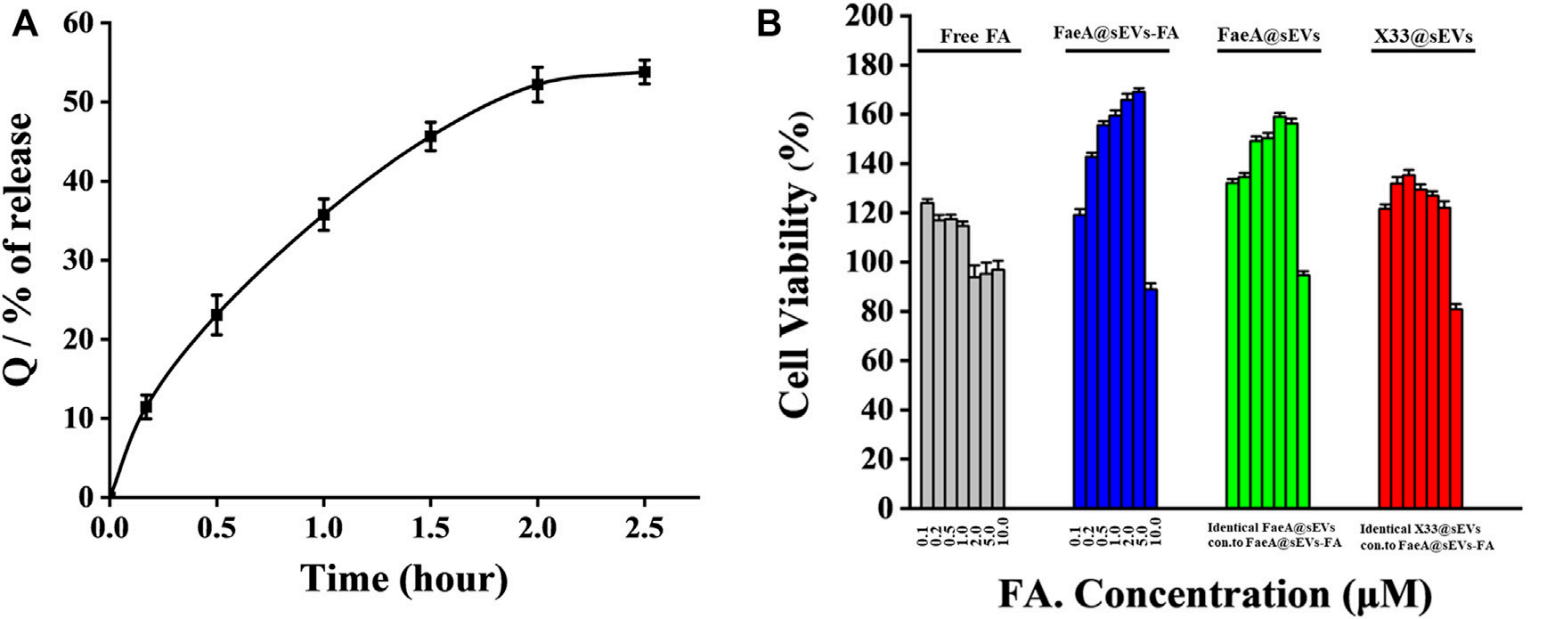
Transdermal administration *in vitro* drug release pattern and cytotoxicity of FaeA@sEVs-FA. **(A)** Cumulative release Q of active drug molecules from FaeA@sEVs-FA within 2.5 h **(B)** CCK-8 based viability measurement of HEK293T cells incubated with free FA, FaeA@sEVs-FA, FaeA@sEVs and X33@sEVs at various concentrations ranging from 0.1 to 10.0 μM, respectively. Note: the particle concentration of X33@sEVs and FaeA@sEVs was identical to FaeA@sEVs-FA. (*n* = 3, mean ± SD).

To further evaluate the potential of FaeA@sEVs-FA in skin repair, we performed *in vitro* drug release assay and cytotoxicity analysis. With *in vitro* drug release curve of FaeA@sEVs-FA was shown in Figure 6A. The curve was fitted with zero-level kinetic equation, first-level kinetic equation, Weibull distribution, Higuchi equation and Ritger-Peppas equation, respectively, and the results were shown in Table 3. The results showed that the first-order kinetic equation and Weibull model fit results were good, and the *in vitro* drug release pattern of the FaeA@sEVs-FA formulation was closest to the Weibull equation, indicating that the FaeA@sEVs-FA formulation has a certain sustained-release effect. This is consistent with the previously reported sEV drug release pattern (Wang et al., 2017; Hu et al., 2021). Previously, the biosafety of *Pichia pastoris* X33-derived sEVs has not been thoroughly examined. The results of the CCK-8 assay showed that X33@sEVs, FaeA@sEVs and FaeA@sEVs-FA all had a wide range of safe concentrations, and could promote the growth of HEK 293T cells within a certain concentration range (Figure 6B). sEV is characterized as nano-sized and biocompatible characteristics (Kutralam-Muniasamy et al., 2015; Henriques-Antunes et al., 2019), which not only improve the stability of FA but also enhance the uptake of FA by cells. Notably, in the FA concentration range of 0.1–1.0 μM, both free FA and FaeA@sEVs-FA showed the promotion of cell proliferation. However, in the FA concentration range of 1.0–5.0 μM, the FaeA@sEVs-FA promoted cell proliferation, which was opposite to the inhibition of cell growth exhibited by free FA. Therefore, in the range of FA concentration of 1.0–5.0 μM, FaeA@sEVs-FA may reverse or alleviate the toxicity of FA to cells, which may be due to the fact that certain nutrients in sEVs interfere with the toxicity of FA to cells. However, when the FA concentration exceeded 5.0 μM, this effect of FaeA@sEVs-FA gradually weakened or even disappeared, which may be due to the too high sEV concentration beyond its safe concentration range.

**TABLE 3:** The regression equation of the release profile of FA from FaeA@sEVs-FA *in vitro*.

Model	Regression equation	*R* ^2^
Zero-order kinetics	*Q* = 21.11t+8.57	0.9013
First-order kinetics	*Q* = 59.27 (1-e^−0.99t^)	0.9959
Higuchi	*Q* = 36.61t^1/2^–1.45	0.9886
Weibull	*Q* = 65.64 (1-e^-(0.79(t+0.0015))^0.87^)	0.9960
Ritger-Peppas	*Q* = 34.77t^0.54^	0.9889

## 4 Conclusion

In summary, inspired by the strategies of active loading of liposomal nanomedicines, pre-drug design and immobilization enzyme, this study developed a high loading capacity and high performance small extracellular vesicle-based drug delivery platform, which is based on esterase-responsive active loading (EAL). The drug loading capacity is significantly better than that of conventional methods. Compared with the classical passive incubation, the EAL can provide a sustainable transmembrane ion concentration gradient and maximize the encapsulation of active molecules into engineered small extracellular vesicles. Furthermore, given the complex heterogeneity of small extracellular vesicles, we designed an isolation method that satisfies the purification of high concentrations of engineered small extracellular vesicles (FaeA@sEVs) from large volumes of cell cultures. Multiple characterization tools were employed to study the physicochemical properties of FaeA@sEVs in depth. Finally, extracellular and intracellular assessments further confirmed its superior performance in terms of slow-release and low toxicity for the sEVs-loaded ferulic acid using the EAL-prepared method. Although a small extracellular vesicle-ferulic acid esterase-ethyl ferulate was used in the study, it should not limit the platform potential for its wide application. In the future, the low loading efficiency of drugs loaded into small extracellular vesicles (sEVs) can be addressed by modifying the inactive part of the target drug with ester bonds *via* various enzymatic reactions. Additionally, this platform suggested that natural components presented in sEVs, such as biological enzymes, may act as “power sources” to facilitate the loading of corresponding active small molecule drugs into sEVs. However, in this experiment, the rough estimation of esterase content in sEVs by enzyme viability assay does not accurately reflect the esterase content in individual FaeA@sEVs obtained by the self-assembling, which poses some difficulties in understanding the self-assembly efficiency. The strategy of fusing target proteins by genetic modification of donor cells and application of membrane localization elements or photo localization elements currently received wide attraction for the engineering of sEVs. The loading efficiency of target esterase should be evaluated if it is applied to the construction of the EAL. In conclusion, we believe that the small extracellular vesicle drug-loading platform based on the EAL in this study complements the existing nanomedicine loading methods, especially to solve the problem of low loading efficiency of water-soluble drugs. The method for separating and purifying high-quality sEVs from large-volume cell cultures provides a feasible tool for the industrial production of sEVs. At the same time, the small extracellular vesicle-loaded ferulic acid offers an improved delivery strategy for its application in anti-aging and skin repair.

## Data Availability

The datasets presented in this study can be found in online repositories. The names of the repository/repositories and accession number(s) can be found below: https://www.ncbi.nlm.nih.gov/, XM_025603867.1.
