# Supplementary material for: Engineered small extracellular vesicles as a versatile platform to efficiently load ferulic acid via an “esterase-responsive active loading” strategy
Source: Front Bioeng Biotechnol. 2022 Nov 9;10:1043130. doi: 10.3389/fbioe.2022.1043130 (PMC9682128; doi:10.3389/fbioe.2022.1043130)
Supplement: Supplementary file 1 [file DataSheet1.docx]

Supplementary Material

**Supplementary Figures and Tables**

**Supporting Materials 1-10:**

**S1:** Construction process of expression plasmid.


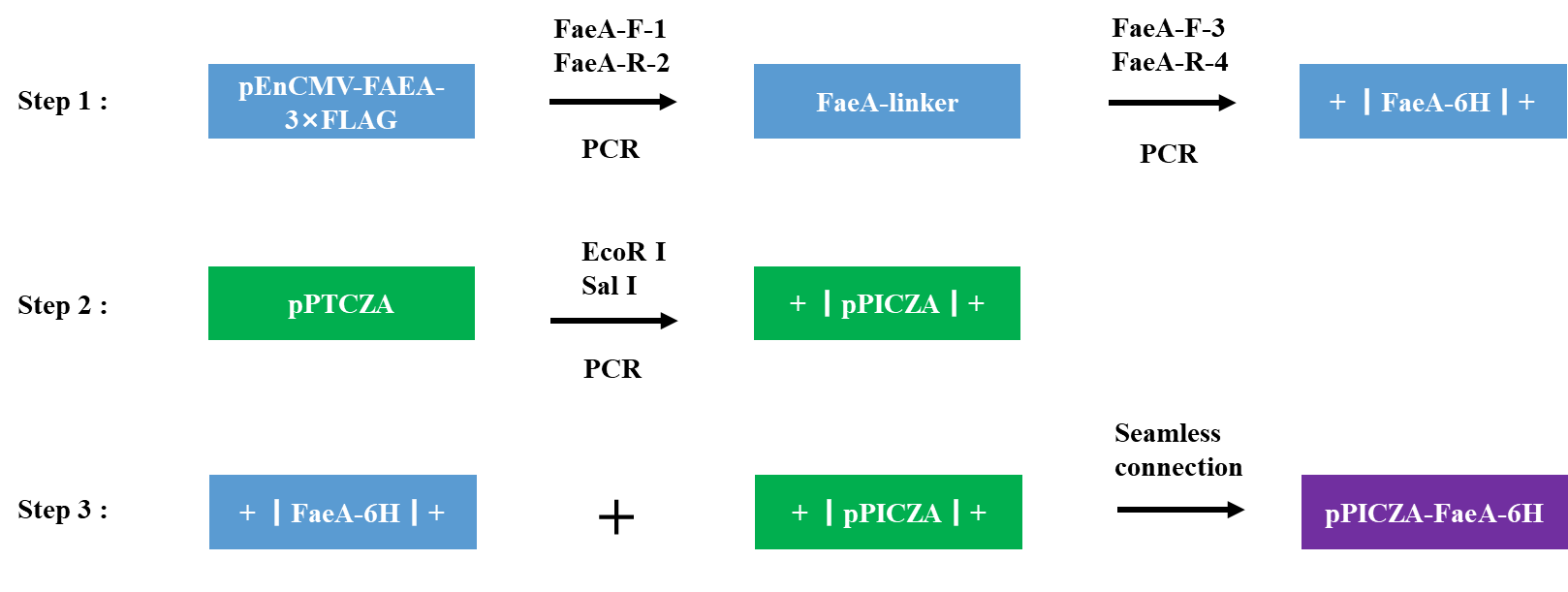


**S2:** Reaction conditions and parameters during construction of expression plasmids.

1. Using the two strands of the pEnCMV-FAEA-3×FLAG plasmid as templates and FaeA-F-1 and FaeA-R-2 as primers, the first round of PCR was performed. The reaction system and conditions were shown in Table S2-1.

Table. S2-1 The first-round PCR reaction

| Reaction system | Volume（μL） | Reaction conditions |  |
| --- | --- | --- | --- |
| ddH_2_O | 33 | Initial denaturation：98℃ 30 s |  |
| 5×Phusion HF Buffer | 10 | Denaturation：98℃ 7 s |  |
| 10 mM dNTPs | 1 | Annealing：61.4℃ 20 s |  |
| FaeA-F-1 | 2.5 | Stretch：72℃ 20 s |  |
| FaeA-R-2 | 2.5 | Cycles：35 times |  |
| Template DNA | 0.5 | Fully stretch：72℃ 7 min |  |
| Phusion Hot Start Ⅱ DNA Ploymerase | 0.5 |  |  |
| Total volume | 50 |  |  |

2. Using the first round of PCR products as templates and FaeA-F-3 and FaeA-R-4 as primers, the second round of PCR reaction was performed. The reaction system and conditions were shown in Table S2-2.

Table. S2-2 The second-round PCR reaction

| Reaction system | Volume（μL） | Reaction conditions |
| --- | --- | --- |
| ddH_2_O | 31.5 | Initial denaturation：98℃ 30 s |
| 5×Phusion HF Buffer | 10 | Denaturation：98℃ 7 s |
| 10 mM dNTPs | 1 | Annealing：65.8℃ 20 s |
| FaeA-F-3 | 2.5 | Stretch：72℃ 30 s |
| FaeA-R-4 | 2.5 | Cycles：35 times |
| Template DNA | 2 | Fully stretch：72℃ 7 min |
| Phusion Hot Start Ⅱ DNA Ploymerase | 0.5 |  |
| Total volume | 50 |  |

3. The expression plasmid pPICZA was double digested by EcoRⅠ and SalⅠ, and incubated at 37℃ for 4 h to complete the digestion. Subsequently, the enzyme was inactivated by heating at 65°C for 20 min, and the digestion system is shown in Table S2-3.

Table. S2-3 The double- endonuclease digestion system

| Reaction system | Volume（μL） | Reaction conditions |
| --- | --- | --- |
| ddH_2_O | 40 | Reaction temperature：37℃ |
| 10×NE Buffer | 5 | Reaction time：4 h |
| EcoRⅠ | 1 | Heat inactivation：65℃ 20 min |
| SalⅠ | 1 |  |
| plasmid | 1 |  |
| Total volume | 50 |  |

4. The recombinant expression plasmids were constructed by ligating the recovered AnfaeA-6H and pPICZA by incubation at 50°C for 30 min according to the Seamless Cloning Kit, and the ligation system is shown in Table S2-4.

Table. S2-4 The ligation system

| Reaction system | Volume（μL） |
| --- | --- |
| pPICZA | 3.88（=100 ng） |
| AnfaeA-6H | 0.76（=88.18 ng） |
| 2×Seamless Cloning Mix | 10 |
| Nuclease free water | 5.36 |
| Total volume | 20 |

**S3:** Results of PCR on recombinant yeast liquid

The yeast liquid PCR was validated using pPICZA universal primers 5'AOX and 3'AOX, and gel electrophoresis analysis showed that the PCR product of X33/pPICZA-FaeA had specific bands at 1100 (lanes 1, 3, 4, and 6), and white spots 1, 3, 4, and 6 were picked. Then, 3, 4 and 6, and sent to Suzhou Jinweiji Biological Co., Ltd. for sequencing, and the sequencing results were as expected. The correctly sequenced recombinant expression plasmid was named recombinant yeast pPICZA-FaeA-6H, indicating that FaeA (843bp) had been successfully integrated into the yeast genome.


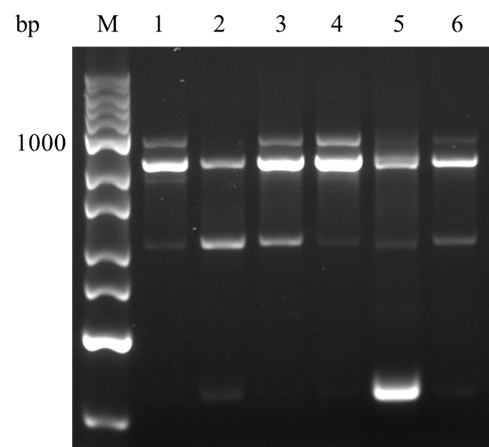


**S4:** Changes in particle number of three sEVs before and after lysis with 0.1% Triton X-100.

| Group | Size (nm) | Concentration (particles/mL) | Number of particles before lysis (particles) | Number of particles after lysis (particles) | Purity |
| --- | --- | --- | --- | --- | --- |
| [X33@sEVs](mailto:X33@sEVs) | 69.48±11.91 | 3.98E+10 | 1.23E+07 | 2.64E+06 | 78.57% |
| [FaeA@sEVs](mailto:FaeA@sEVs) | 64.46±10.93 | 1.21E+11 | 2.24E+07 | 2.79E+06 | 87.54% |
| [FaeA@sEVs-FA](mailto:FaeA@sEVs-FA) | 70.01±10.92 | 3.64E+10 | 1.69E+07 | 2.21E+06 | 86.87% |

**S5:** Western blot image of sEV loaded with ferulic acid esterase.


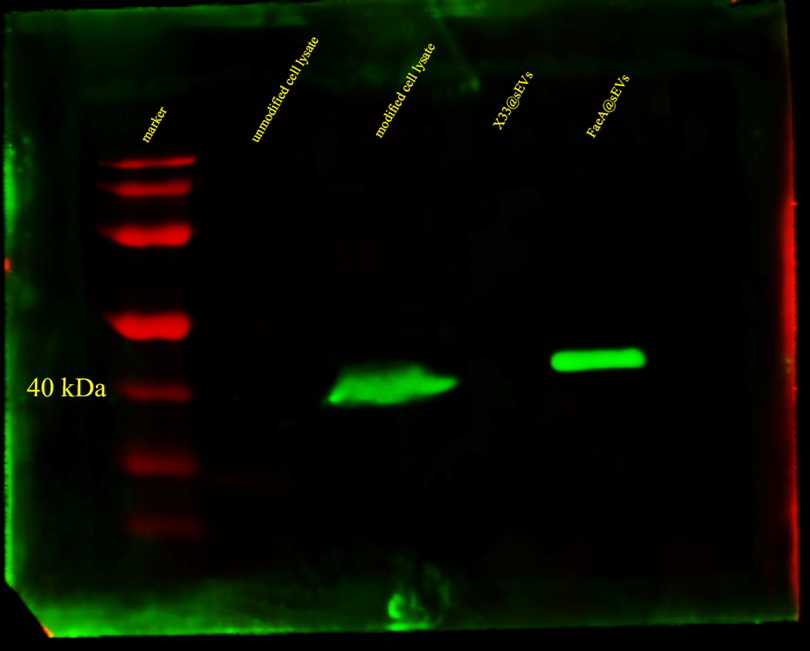


**S6:** The particle size distribution diagrams of three sEVs.

**
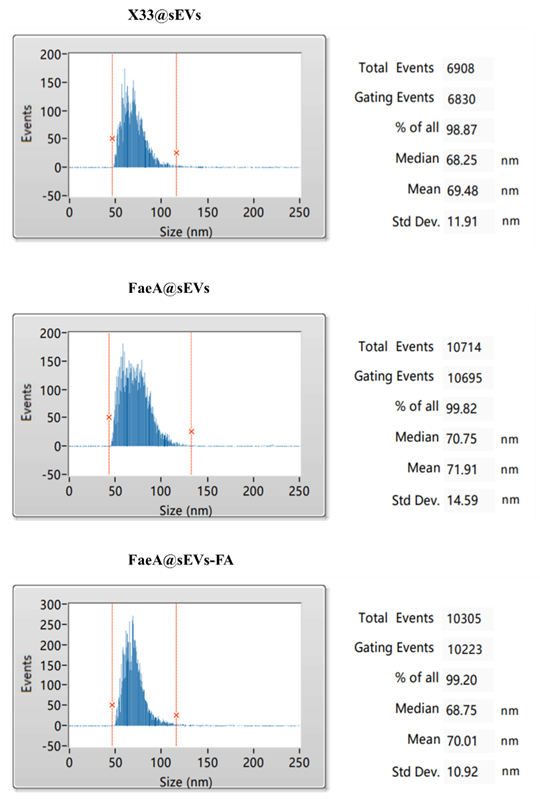
**

**S7:** Summary of characterization data of three sEVs.

|  | Characterization index | X33@sEVs | FaeA@sEVs | FaeA@sEVs-FA |
| --- | --- | --- | --- | --- |
| Nano-flow cytometry | Particle size (nm) | 69.48±11.91 | 71.91±14.59 | 70.01±10.92 |
|  | Concentration Counting (particles/mL) | 3.98E+10 | 1.21E+11 | 3.64E+10 |
|  | Purity (%) | 78.57 | 87.54 | 86.87 |
| Malvern particle size analyzer | Particle size (nm) | 137.00±42.50 | 205.50±53.69 | 193.00±39.31 |
|  | Zeta potential (mV) | －11.20±10.60 | －13.20±15.70 | －17.70±14.60 |
|  | PDI | 0.330 | 0.303 | 0.400 |
| BCA kit | Protein concentration (μg/mL) | 450.00 | 400.00 | 350.00 |

**S8:** Chromatogram of EF and FA standard.


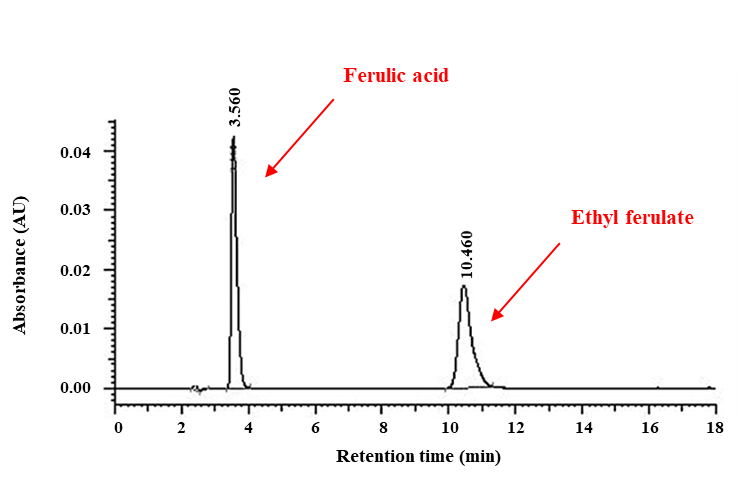


**S9:** The drug loading efficiency of EF in engineered sEVs at different concentrations of EF

To 100 μL 0.400 mg/mL of engineered sEVs, 100 μL different concentrations of EF was added, so that the mass ratios of engineered sEVs to EF in the mixed system were 4:1, 2:1, 1:1, 1:1.5, and 1:2. 800 μL PBS (0.1×, pH 4.0) was added to the above mixed system to form the reaction system, respectively. As the EF is hydrolyzed to ferulic acid, the loading efficiency of the pre-drug model drug will eventually be calculated in the form of FA, and the assay procedure is described in Method 2.11.

Table. S9 The drug loading efficiency of EF in engineered sEVs at different concentrations of EF

| EF (μg/mL) | Drug loading (μg/40 μg engineered sEVs) | Encapsulation efficiency % |
| --- | --- | --- |
| 10 | 9.22±0.89 | 42.20±4.06 |
| 20 | 12.14±0.91 | 27.79±2.09 |
| 40 | 25.24±3.13 | 28.88±3.58 |
| 60 | 36.97±3.93 | 28.21±3.00 |
| 80 | 54.10±7.47 | 30.96±4.28 |
|  | | |


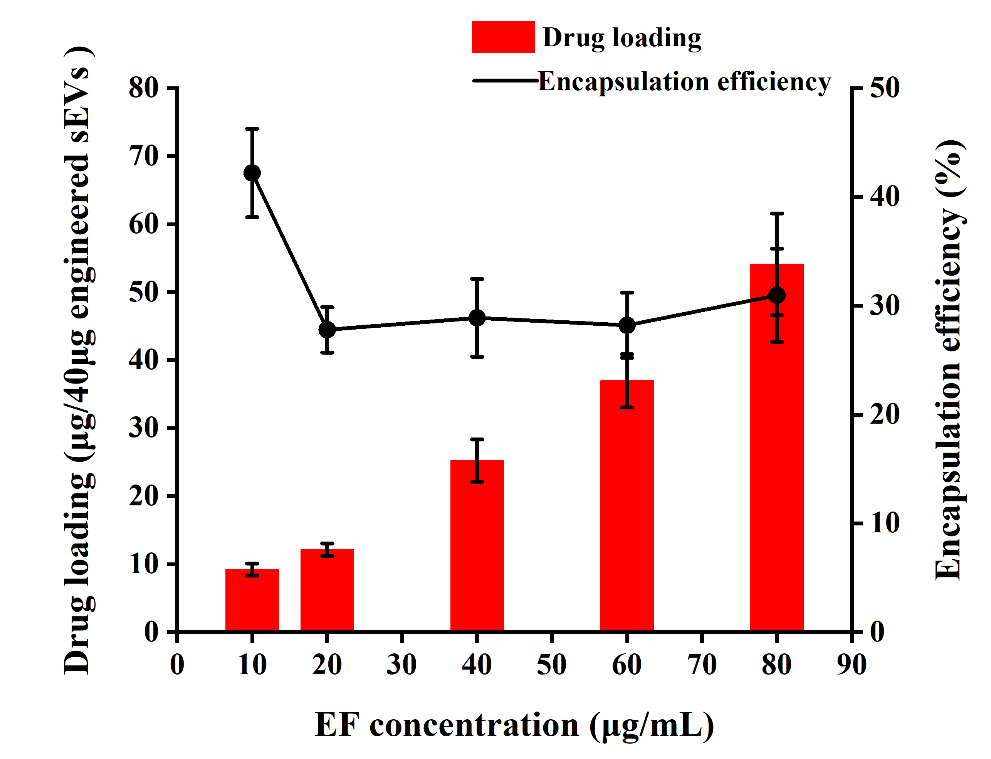


Figure. S9 The drug loading efficiency of EF in engineered sEVs at different concentrations of EF

**S10:** The drug loading efficiency of EF in engineered sEVs. b/a=1.16, indicating that the average amount of FA encapsulated in individual FaeA@sEVs was 1.16 folds higher than that of individual X33@sEVs.

| Group | Drug loading (μg/40 μg sEVs) | Drug loading (μg/40 μg sEVs) | Drug loading (μg/individual sEV) | Encapsulation efficiency (%) |
| --- | --- | --- | --- | --- |
| X33@sEVs-FA | 9.92±1.28 | 9.92±1.28 | (5.54E-13)±(7.13E-14)^a^ | 7.18±0.92 |
| FaeA@sEVs-FA | 54.10±7.47** | 54.10±7.47** | (6.40E-13)±(3.30E-14)^b^ | 30.96±4.28** |
| X33@sEVs-#FA | 22.19±3.18 | 22.19±3.18 | (1.24E-12)±(1.78E-13) | 16.07±2.31 |
| FaeA@sEVs-#FA | 29.39±5.15 | 29.39±5.15 | (6.07E-13)±(1.06E-13) | 16.82±2.95 |
